# A Critical Analysis of the Exercise Prescription and Return to Activity Advice That Is Provided in Patient Information Leaflets Following Lumbar Spine Surgery

**DOI:** 10.3390/medicina55070347

**Published:** 2019-07-07

**Authors:** Matthew Low, Louise C. Burgess, Thomas W. Wainwright

**Affiliations:** 1 Therapy Outpatient Department, The Royal Bournemouth and Christchurch Hospitals NHS Foundation Trust, Bournemouth BH7 7DW, UK; 2 Orthopaedic Research Institute, Bournemouth University, Bournemouth BH8 8EB, UK

**Keywords:** patient education, patient information leaflets, lumbar spine surgery, enhanced recovery after surgery (ERAS), rehabilitation, activities of daily living (ADL)

## Abstract

*Background and objectives:* Lumbar spine surgery may be considered if pharmacologic, rehabilitation and interventional approaches cannot provide sufficient recovery from low back-related pain. Postoperative physiotherapy treatment in England is often accompanied by patient information leaflets, which contain important rehabilitation advice. However, in order to be an effective instrument for patients, the information provided in these leaflets must be up to date and based on the best available evidence and clinical practice. This study aims to critically analyse the current postoperative aspects of rehabilitation (exercise prescription and return to normal activity) that are provided in patient information leaflets in England as part of an evaluation of current practice following lumbar spine surgery. *Materials and Methods:* Patient information leaflets from English National Health Service (NHS) hospitals performing lumbar spine surgery were sourced online. A content analysis was conducted to collect data on postoperative exercise prescription and return to normal activities. *Results:* Thirty-two patient information leaflets on lumbar surgery were sourced (fusion, *n* = 11; decompression, *n* = 15; all lumbar procedures, *n* = 6). Many of the exercises prescribed within the leaflets were not based on evidence of clinical best practice and lacked a relationship to functional activity. Return to normal activity advice was also wide ranging, with considerable variation in the recommendations and definitions provided. *Conclusions:* This study highlights a clear variation in the recommendations of exercise prescription, dosage and returning to normal activities following lumbar spine surgery. Future work should focus on providing a consistent and patient-centred approach to recovery.

## 1. Introduction

Low back pain continues to be ranked as the leading cause of years lived with disability in the world [1] and radicular leg pain has been reported to have a lifetime incidence between 13 and 40% [2]. The treatments for patients with low back pain and/or sciatica include pharmacologic, rehabilitation and interventional (injection therapy) approaches. If such approaches fail to provide sufficient recovery, then lumbar spine surgery may be considered. Surgery to the spine may involve decompression (the surgical removal of structures of the spine felt to be compressing neural structures) and fusion (surgical stabilisation of vertebral segments) or both procedures to one or more levels of the lumbar spine. Following spine surgery, effective postoperative rehabilitation is considered important to help patients return to normal function and achieve their recovery goals [3].

However, there appears to be significant variation in the delivery of postoperative spine rehabilitation in the United Kingdom (UK), with a variability of practice, mode of delivery, and with a limited use of outcome measures [4]. Perioperative physiotherapy has been reported as inconsistent [5], with no uniformity regarding advice on activities of daily living, and a lack of structured outcome evaluation [6]. The reason for this inconsistency may be due to the complex multidimensional nature of low back-related pain that encompasses a bio-psychosocial context, and as such there is limited evidence to support the effectiveness of one particular rehabilitation intervention over another [7,8]. In addition, physical activity levels are reported to remain low following lumbar spine surgery [9] despite the importance of physical exercise in decreasing the long-term risk of other lifestyle co-morbid conditions such as cardiovascular disease, diabetes and obesity.

Enhanced Recovery after Surgery (ERAS) programmes are being increasingly applied to spinal surgery and there is emerging evidence for the inclusion of ERAS components within surgical pathways [10]. Given that the aim of ERAS is to accelerate the recovery process, through standardised, multidisciplinary surgical pathways, it is pertinent to look judiciously at current postoperative physiotherapy treatment interventions to ensure that they are both safe and efficacious following lumbar spine surgery. Postoperative physiotherapy treatment is often accompanied by educational material in the form of patient information leaflets. They are provided to patients with the aim of providing important consumer health information in an easy to read format. Although information leaflets may improve patients’ knowledge, satisfaction and adherence to treatment [11], their quality and effectiveness has been criticised [12], and a lack of adherence to ERAS principles has been highlighted in orthopaedic resources [13]. Patient leaflets can be used to disseminate important postoperative rehabilitation information which can be difficult to retain during face-to-face meetings with clinicians. However, in order to be an effective tool for patients, the information provided in the leaflets must be up to date and based on evidence of best clinical practice.

Preliminary work has evaluated the overall quality of patient leaflets for lumbar spine surgery [14] and, therefore, the aim of this study is to critically analyse the current postoperative aspects of rehabilitation (exercise prescription and return to activity) that are provided in patient information leaflets in England as part of an evaluation of current practice.

## 2. Materials and Methods

This study was conducted by adopting a qualitative data collection method which informed a quantitative data analysis procedure, a method previously used to evaluate the content of patient information leaflets [13]. Patient information leaflets from English National Health Service (NHS) hospitals performing lumbar spine surgery were sourced, and then a content analysis was conducted to collect relevant data from these resources. The collected data were organised into basic descriptive statistics in preparation for analysis and discussion. All data were collected from freely available content available from hospital websites and ethical approval was not pursued as this study did not involve human participation. The multi-stage data extraction process is portrayed in Table 1.

### 2.1. Search Strategy

To identify patient information leaflets, a full list of English NHS hospitals that offer lumbar fusion, laminectomy and discectomy was sourced from the NHS “find a procedure” online tool (https://www.nhs.uk/Service-Search/Hospital/LocationSearch/8/Procedures). Each hospital website was then examined thoroughly in an attempt to source patient information leaflets. It was found that not all hospitals offering the procedures provided patient information leaflets that were available to patients online and, in some cases, a number of hospitals or NHS trusts shared the same resource. In such cases, only one version of the leaflet was downloaded and analysed.

### 2.2. Eligibility Criteria

Patient information leaflets were only included within the study if they met the eligibility criteria listed in Table 2. Patient information leaflets on other types of spine surgery were excluded due to the specific objectives of the study. The leaflets were included if they were either (i) patient information, (ii) exercise prescription or (iii) both patient information and exercise prescription. The recent National Institute of Clinical Excellence (NICE) [15] guidelines for low back pain and sciatica for patients over 16’s advise to not offer spinal fusion for people with low back pain unless as part of a randomised controlled trial and also to not offer disc replacement in people with low back pain. The study therefore excludes lumbar disc replacement for low back pain but includes lumbar spine fusion with decompression surgery in conjunction with lumbar decompression surgery. The analysis of patient information has been separated into three categories: lumbar fusion surgery, lumbar decompression surgery (laminectomy and discectomy procedures), and all lumbar procedures (where the hospital has not differentiated between rehabilitation programmes for decompression and fusion surgery).

### 2.3. Pilot Testing

To develop a suitable data extraction procedure, a sample of five patient information leaflets were sourced and used for pilot testing. A flowchart was created which addressed various elements of the postoperative rehabilitation advice listed within patient information leaflets, for example: exercise prescription, walking and stair climbing advice, return to sport and return to activities of daily living. Two members of the research team (LB and ML) reviewed the flowchart individually and discussed any changes that needed to be made to the methodology.

### 2.4. Evaluation

**Figure 1:**
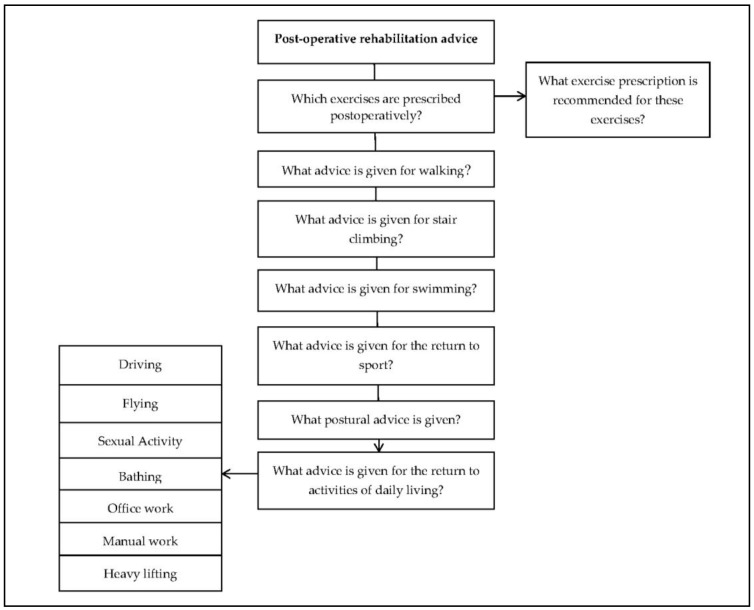
Data extraction flow chart.

A content analysis was then used to collect data from the patient information leaflets, informed by previous studies that have evaluated patient information [13,16,17]. Each patient information leaflet was systematically reviewed by two researchers (LB and ML) and the data was recorded in a Microsoft Excel (2010) spreadsheet. Data were organised into percentage totals to allow the comparison of basic descriptive statistics, and a thematic analysis was conducted to compare the content of patient information leaflets. Due to the limited data available, no statistical analyses were performed. Minor additions were made to the original flowchart, with the final version shown in Figure 1.

## 3. Results

Thirty-two patient information leaflets were sourced from English NHS hospitals or trusts (Supplementary Materials Table S1). Leaflets were grouped into either (i) lumbar fusion surgery (*n*=11), (ii) lumbar decompression surgery (laminectomy, discectomy, and microdiscectomy) (*n* = 15) or (iii) all lumbar surgeries (fusion and decompression) (*n* = 6). Only 16 (50%) of the leaflets included advice on postoperative exercise prescription.

### 3.1. Exercises

Fourteen exercises were identified from the information leaflets that are given to patients across the NHS hospitals in England after lumbar spine surgery and were broadly identified into four themes. The main themes of the exercises were: (i) isometric muscle activation (ii) range of movement, (iii) neurodynamic and (iv) movement control exercises. Examples of these exercises vary in the literature and therefore descriptions of the four main themes are listed in Table 3. One leaflet recommended strengthening in a functional weight bearing position following decompression surgery (sit to stand). One of the exercises could be described as a circulatory exercise as well as a range of movement exercise (ankle pump). All the included exercises and themes are listed in Table 4 alongside how frequently they were identified. The most common exercise prescribed following spinal surgery was the muscle activation exercise focusing on transversus abdominus with the patient lying in a supine position (47%). The literature also described this as a motor control exercise [18,19,20,21] with it being used in almost half of the selected patient information leaflets.

The second most common exercise was a range of movement exercise described as knee rolls (34%) involving the patient rolling their knees from side to side in a supine position. This exercise appeared in a third of the sourced leaflets. In around a quarter of the patient information leaflets, isometric gluteal exercises given in the supine position (27%), ankle pump exercises were given in the supine position (27%) and neurodynamic exercises were recommended in a sitting position (27%).

### 3.2. Dosage of Exercise Prescription

Ten leaflets specified the dosage of exercises with the most common prescription recorded as three sets of ten repetitions across all exercises. Some exercise sheets were left blank for the physiotherapist to complete, presumably on discharge from hospital; however, it was not clear what assessment or clinical reasoning took place in order to provide this information. Some leaflets did not provide any dosage information and three were left for the patient to decide themselves, with advice such as “repeat as you feel able”, “five to ten times at regular intervals” and “repeat five times.”

### 3.3. Return to “Normal” Activities

**Figure 2:**
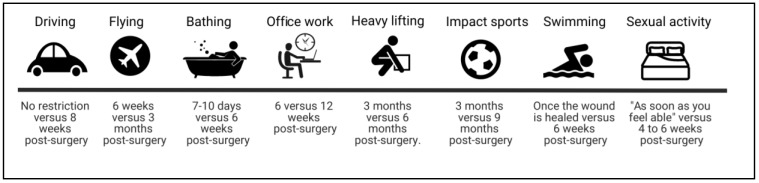
Examples of variation in advice on return to “normal” activities after lumbar fusion surgery.

Return to normal activity advice was also wide ranging, with considerable variation in the recommendations and definitions provided in patient information leaflets. The variation in advice for returning to normal activities following lumbar surgery has been shown in Table 5 and Figure 2. It was also noted that only 15 out of 32 (47%) leaflets provided information on the expected length of stay in hospital. Most leaflets encouraged patients to return to walking as soon as possible after surgery, but only one provided specific walking targets with clear advice on progression [23].

### 3.4. Recognition of Uncertainty

A number of the leaflets recognised that returning to certain normal activities required further advice presumably following a re-assessment—examples include asking the surgical team at a follow up appointment on advice on returning to work, while other leaflets suggest requesting advice from a general practitioner (GP). More commonly, patients were advised to discuss activities that involve a greater perceived risk (for example, return to manual work or high impact activities) directly with their surgeon.

## 4. Discussion

The findings of this study are similar to others [4,6], whereby there is a significant amount of inconsistency and variation in advice provided across the majority of domains of clinical practice and patient management. Variation exists in the prescription of exercise, the advice to return to normal activities and also returning to work. It is reasonable to expect uncertainty, particularly in the context of co-morbidities and premorbid conditions. However, these elements were not explicitly discussed in any of the patient information leaflets. One could argue that this finding is hardly surprising given the complexity and multi-dimensional nature of patients that present with low back and/or radicular pain. The literature is clear that using a bio-psychosocial [24,25,26] perspective is advantageous, particularly in this cohort of patients. However, the lack of advice on the engagement in social activities as well as the recognition of the potential psychological burdens of recovery after surgery, including cases of post-traumatic stress [27,28], is conspicuous by its absence.

It may be argued that the prescription of exercise detailed within the leaflets lacks critical insight, and this is highlighted by the commonly prescribed transversus abdominus exercise in supine. Many well-conducted studies now question the effectiveness of transversus abdominus exercises in back pain populations [20,29] and there is a suggestion of the possibility of facilitating higher levels of fear avoidance [20] in regard to activity. Given that the premise of the transverse abdominus exercise is to preferentially activate the deeper ‘stabilising’ muscles of the spine and therefore reduce the proposed mechanical clinical instability of the spine [18,19], it is interesting to evaluate their use after spinal stabilisation surgery. Once the spine has been surgically fixated, it may be argued unnecessary to try and achieve further stabilisation by activating the deeper stabilising muscles of the spine. Kang et al. [30] compared general exercise, extension based exercise and a specific stabilising muscle exercise group in an 8-week programme, three months after lumbar fusion. The study found that all of the exercise groups improved with respect to pain, disability, strength and scores using a pressure biofeedback unit (that may indicate improved deeper spinal muscle function [31]). There were no significant differences between the groups. The spinal stabilising group were found to have a non-statistically significant improvement in pressure biofeedback function but did not outperform the other exercises with respect to pain or disability.

It may also be observed that the most commonly prescribed exercises were not functional, in that they did not represent movements that could directly impact on function, with the exception of one exercise leaflet that recommended the sit-to-stand movement. The aim and purpose of providing range of movement exercises, such as lumbar rotation in lying, which is limited mechanically in the lumbar spine, immediately following spinal surgery is questionable, particularly in the context of spinal fusion. The aim may be to mobilise the hips or upper trunk. However, there are many other methods to perform this such as in standing or in other weight bearing, functional positions. The majority of exercises were bed exercises which are limited with the transferability to function. In addition, the dosages of exercises were not explicit in their reasoning or premise which is in keeping with a systematic review of the rehabilitation literature [32].

The apparent indiscriminate recommendation of neurodynamic exercises for radicular pain without judicious patient selection is also of interest. The rationale of such exercises is aimed at restoring the homoeostasis in and around the nervous system by mobilisation of the nervous system itself, or the structures that surround it [22]. Their use as a prescriptive exercise is questionable given the lack of consistency and high risk of bias within the evidence base [22] and that they have been shown to not have a statistical or clinically meaningful effect [33].

The significant variation of information provided to patients on the return to normal activities was perhaps not surprising given the findings from other procedures [13], and highlights the need to gain consensus amongst clinicians and provide explicit explanations on the reasons behind the activity restrictions. It may be due to soft tissue healing times or perhaps bone healing times for fusion surgery. In such cases, a question is raised regarding why such variation exists unless it is due to co-morbidities that affect or delay healing, for example, diabetes or age-related reasons. However, those reasons were not demonstrated in the patient information leaflets we sourced and may suggest either a lack of understanding or consideration in their development. A recent personal narrative account of recovery, written by a GP recovering from spinal surgery, also highlights the shortfall in a consensus on rehabilitation [34], and poses the question, that if a recognised medical healthcare professional found postoperative information challenging, confusing and contradictory, how will non-health care professionals feel?

The recent “Get It Right First Time” (GIRFT) national report on spinal services also highlights the significant variation in provision and outcomes across spinal services in the UK [35]. The report welcomes and suggests recommendations to reduce variability, and as such, the further implementation of ERAS may help to reduce the variation in surgical pathways, as it has done in other procedures [36]. Standardised ERAS pathways not only improve surgical outcomes but also make it easier to provide more detailed information to patients prior to their operation regarding their recovery, because of the increased certainty of both process and outcome. Therefore, the emergence of applying ERAS principles to spinal pathways [10] provides an ideal opportunity to reconsider and improve the provision of exercise and return to activity instructions.

### Limitations

As a previously highlighted limitation with studies evaluating patient information leaflets [13,14], the information provided may not accurately reflect clinical practice following lumbar spine surgery, and it may be that patients are offered additional support to the information provided in these resources. Our study is also limited as it does not include a resource from every English NHS hospital that provides lumbar spine surgery, but instead just those that were available to download online. Finally, the leaflets available online may not replicate the resources given in hospital to patients.

## 5. Conclusions

This study highlights a clear variation in the recommendations of exercise prescription, dosage and returning to normal activities following lumbar spine surgery. The exercises prescribed within patient information leaflets lack a relationship to functional activity and their effectiveness may be questioned. The exercises also appear to lack clarity with respect to dosage and aims towards patient centred outcomes. The activity restrictions given in the patient information leaflets are not explicit in their reasoning and may induce fear and anxiety in patients following lumbar spine surgery, all of which may adversely affect post-surgical outcomes. This may perhaps be even more significant given that there is such a wide variation between NHS trusts.

Furthermore, the multi-dimensional nature of treating patients with low back and/or radicular leg pain does not appear to be represented in the information given to patients following spinal surgery including the importance of psychological and social support. There is a pressing need to evaluate the information provided for patients undergoing spinal surgery at English NHS hospitals with respect to the rehabilitation and restoration of normal activities. Future work should focus on providing a consistent and patient-centred approach to recovery.

## Figures and Tables

**Table 1:** Multi-stage data extraction process.

Process
The NHS Choices webpage was searched for hospitals that provide lumbar fusion, laminectomy or discectomy procedures. Hospital websites were searched for patient information leaflets. Thirty-two lumbar spinal surgery patient information leaflets were sourced. Patient information leaflets were assessed for eligibility. A review of five leaflets was conducted to create a flowchart for content analysis. The flow chart was pilot tested on a sample of patient information leaflets.. The revised flowchart was used to extract data from all of the patient information leaflets. Data were organised into basic descriptive statistics for analysis.

**Table 2:** Eligibility criteria for inclusion.

Inclusion	Exclusion
Patient	
Lumbar spinal fusion patients (all surgical techniques) for back and leg pain or for the treatment for low back pain following previous decompression surgery for predominant leg pain. Lumbar decompressions surgery (laminectomy, discectomy) for predominant leg pain. Patients aged over 16 years.	Any other spine surgery (lumbar disc replacement, correction of spinal deformity, removal of spinal tumours). Patients under 16 years old.
Information	
Postoperative physiotherapy. Exercise prescription. Home rehabilitation programmes. Return to normal activity advice.	
Source	
English NHS Hospital.	Independent Providers of Healthcare. Charity or Research Institute Information Blogs.
Format	
PDF or Word Document. Hospital provided webpage. Latest version.	Archived versions.

**Abbreviations:** NHS; National Health Service; PDF, portable document format.

**Table 3:** Definition, aims and examples of exercise themes.

Theme	Definition and Aims	Examples
Isometric muscle activation	A theme of exercises whereby the muscle length stays the same during contraction. Usually, this exercise is given to reduce joint motion while still achieving a level of recruitment of muscle activity. It is usually given to either protect joint loading or to isolate individual muscle contractions.	Transversus abdominus in supine, isometric quadriceps, isometric gluteal and pelvic floor exercises
Range of movement	A theme of exercises that intends to provide movement across a specified range to the joint region. This can be across one or more joints depending on the area specified.	Knee rolls, hip flexion, extension, abduction, trunk side bends, ankle dorsi/plantarflexion and pelvic tilting
Neurodynamic	A theme of exercises that aims at restoring the homoeostasis in and around the nervous system by mobilisation of the nervous stem itself or the structures that surround the nervous system [22].	Seated knee flexion/extension and hamstring stretch
Movement control	A theme of exercises that intends to produce specific restraints to joint or body motion in order to reduce ‘aberrant’ joint movement or body motion.	Side lying hip abduction (clam), supine bent knee fall out

**Table 4:** Identified postoperative exercises, theme and frequency of provision.

Exercise	Theme	Frequency n (%)
Transverse abdominus	Isometric muscle activation	7 (47%)
Knee rolls	Range of movement	5 (34%)
Static glutes	Isometric muscle activation	4 (27%)
Ankle pumps	Range of movement	4 (27%)
Neurodynamic exercises	Neurodynamic	4 (27%)
Pelvic tilts	Range of movement	3 (20%)
Hip flexion	Range of movement	3 (20%)
Static quadriceps	Isometric muscle activation	2 (13%)
Hip abduction (bent knee fall out)	Movement control	2 (13%)
Standing side bends	Range of movement	1 (7%)
Hip extension	Range of movement	1 (7%)
Side-lying clam exercise	Movement control	1 (7%)
Hamstring stretch	Range of movement	1 (7%)
Heel raises	Range of movement	1 (7%)
Sit to stand	Functional Strength	1 (7%)

**Table 5:** Examples of variation in advice on return to ‘normal’ activities after lumbar fusion and decompression surgery.

Activity	Examples of Range of Advice for Return to “Normal” Activity	
	Fusion surgery	Decompression surgery
Driving	No restriction versus 8 weeks post-surgery	24 hours versus 6 weeks post-surgery
Flying	6 weeks versus 3 months post-surgery	2 weeks versus 2 months
Sexual activity	“As soon as you feel comfortable” versus 4–6 weeks post-surgery	“As soon as you feel comfortable” versus 3–4 weeks post-surgery
Bathing	7–10 days versus 6 weeks post-surgery	No restriction if wound is protected versus 6 weeks post-surgery
Office work	6 versus 12 weeks post-surgery	2 weeks versus 8 weeks post-surgery.
Manual Work	6 weeks versus 6 months post-surgery	6 weeks versus 6 months post-surgery
Heavy lifting	Definition of heavy lifting: 10 kg. Return to heavy lifting: 3 months versus 6 months post-surgery.	Definition of heavy lifting: 1 kg (half a full kettle) versus 10 kg Return to heavy lifting: 4–6 weeks post-surgery.
Swimming	Once the wound is healed versus 6 weeks after surgery.	Straight after surgery versus 6 weeks post-surgery.
High impact sports	3 months versus 9 months post-surgery	4 weeks versus 6 months post-surgery
Posture	Vary posture every 20 min versus every 40 min	Vary posture every 20 min versus every 60 min
Discharge home	Expected discharge on day 2 following surgery versus 5–7 days.	Expected discharge on the day of surgery versus 5–7 days after surgery.

## Supplementary Materials

The following are available online at https://www.mdpi.com/1010-660X/55/7/347/s1, Table S1: Included hospitals/NHS trusts.
