# Supplementary material for: A Critical Analysis of the Exercise Prescription and Return to Activity Advice That Is Provided in Patient Information Leaflets Following Lumbar Spine Surgery
Source: Medicina (Kaunas). 2019 Jul 7;55(7):347. doi: 10.3390/medicina55070347 (PMC6681378; doi:10.3390/medicina55070347)
Supplement: Supplementary file 1 [file medicina-55-00347-s001.pdf]

Table 1. Included Hospitals/NHS Trusts.

	Hospital	Procedure (s)
1	Brighton and Sussex University Hospitals NHS Trust	Lumbar spinal surgery
2	Cambridge University Hospitals NHS Foundation Trust	Lumbar spinal surgery
3	Derby Teaching Hospitals NHS Foundation Trust	Posterior lumbar fusion
4	Gateshead Health NHS Foundation Trust	Lumbar spinal fusion
5	Guy's and St Thomas' NHS Foundation Trust	Anterior and posterior lumbar fusion surgery
6	Guy's and St Thomas' NHS Foundation Trust	Primary and revision discectomy
7	King's College Hospital NHS Foundation Trust	Lumbar spinal surgery
8	Musgrove Park Hospital	Lumbar spinal fusion
9	Norfolk and Norwich University Hospitals NHS Foundation Trust	Microdiscectomy or decompression
10	North Bristol NHS Trust	Lumbar spinal fusion
11	North Bristol NHS Trust	Discectomy, microdiscectomy, spinal decompressions
12	Nottingham University Hospitals NHS Trust	Lumbar spinal fusion/disc replacement
13	Nottingham University Hospitals NHS Trust	Lumbar Spine Decompression/Discectomy
14	Oxford Radcliffe Hospitals NHS Trust	Lumbar discectomy, microdiscectomy and decompressive laminectomy
15	Royal Berkshire NHS Foundation Trust	Lumbar spinal fusion
16	Royal Surrey County Hospital NHS Foundation Trust	Lumbar spinal surgery
17	Royal United Hospitals Bath NHS Trust	Microdiscectomy/spinal stenosis decompression
18	Salisbury NHS Foundation Trust	Lumbar spinal fusion
19	Salisbury NHS Foundation Trust	Discectomy/Decompression
20	South Tees Hospitals NHS Foundation Trust	Lumbar spinal fusion
21	South Tees Hospitals NHS Foundation Trust	Discectomy
22	South Tees Hospitals NHS Foundation Trust	Decompression
23	Southend University Hospital NHS Foundation Trust	Lumbar spinal surgery
24	St George's Healthcare NHS Trust	Microdiscectomy/laminectomy
25	The Dudley Group NHS Foundation Trust	Microdiscectomy
26	The Ipswich Hospital NHS Trust	Anterior and posterior lumbar fusion

27	The Royal Liverpool and Broadgreen University Hospitals NHS Trust	Lumbar spinal surgery
28	University Hospital of North Staffordshire	Discectomy/decompression
29	University Hospitals Birmingham NHS Foundation Trust	Discectomy
30	West Hertfordshire Hospitals NHS Trust	Lumbar spinal fusion
31	West Hertfordshire Hospitals NHS Trust	Discectomy
32	West Hertfordshire Hospitals NHS Trust	Decompression

Abbreviations: NHS, National Health Service.
